# Sodium phenylbutyrate inhibits Schwann cell inflammation via HDAC and NFκB to promote axonal regeneration and remyelination

**DOI:** 10.1186/s12974-021-02273-1

**Published:** 2021-10-16

**Authors:** Anjali Yadav, Tzu-Chieh Huang, Szu-Han Chen, Thamil Selvee Ramasamy, Yuan-Yu Hsueh, Shau-Ping Lin, Fu-I Lu, Ya-Hsin Liu, Chia-Ching Wu

**Affiliations:** 1 Taiwan International Graduate Program in Interdisciplinary Neuroscience, Academia Sinica, Taipei, Taiwan; 2 Institute of Clinical Medicine, College of Medicine, National Cheng Kung University, Tainan, Taiwan; 3 Department of Cell Biology and Anatomy, College of Medicine, National Cheng Kung University, Tainan, Taiwan; 4 Institute of Basic Medical Sciences, College of Medicine, National Cheng Kung University, Tainan, Taiwan; 5 International Center for Wound Repair and Regeneration, National Cheng Kung University, Tainan, Taiwan; 6 Division of Plastic and Reconstructive Surgery, Department of Surgery, College of Medicine, National Cheng Kung University Hospital, National Cheng Kung University, Tainan, Taiwan; 7 Stem Cell Biology Laboratory, Department of Molecular Medicine, Faculty of Medicine, University of Malaya, Kuala Lumpur, Malaysia; 8 Institute of Biotechnology, College of Bio-Resources and Agriculture, National Taiwan University, Taipei, Taiwan; 9 Department of Biotechnology and Bioindustry Sciences, College of Bioscience and Biotechnology, National Cheng Kung University, Tainan, Taiwan; 10 Department of Life Sciences, College of Bioscience and Biotechnology, National Cheng Kung University, Tainan, Taiwan; 11 Department of Biomedical Engineering, National Cheng Kung University, Tainan, Taiwan

**Keywords:** Inflammation, HDAC inhibitor, Peripheral nerve injury, Schwann cells, Regeneration and myelination

## Abstract

**Background:**

Epigenetic regulation by histone deacetylases (HDACs) in Schwann cells (SCs) after injury facilitates them to undergo de- and redifferentiation processes necessary to support various stages of nerve repair. Although de-differentiation activates the synthesis and secretion of inflammatory cytokines by SCs to initiate an immune response during nerve repair, changes in either the timing or duration of prolonged inflammation mediated by SCs can affect later processes associated with repair and regeneration. Limited studies have investigated the regulatory processes through which HDACs in SCs control inflammatory cytokines to provide a favorable environment for peripheral nerve regeneration.

**Methods:**

We employed the HDAC inhibitor (HDACi) sodium phenylbutyrate (PBA) to address this question in an in vitro RT4 SC inflammation model and an in vivo sciatic nerve transection injury model to examine the effects of HDAC inhibition on the expression of pro-inflammatory cytokines. Furthermore, we assessed the outcomes of suppression of extended inflammation on the regenerative potential of nerves by assessing axonal regeneration, remyelination, and reinnervation.

**Results:**

Significant reductions in lipopolysaccharide (LPS)-induced pro-inflammatory cytokine (tumor necrosis factor-α [TNFα]) expression and secretion were observed in vitro following PBA treatment. PBA treatment also affected the transient changes in nuclear factor κB (NFκB)-p65 phosphorylation and translocation in response to LPS induction in RT4 SCs. Similarly, PBA mediated long-term suppressive effects on HDAC3 expression and activity. PBA administration resulted in marked inhibition of pro-inflammatory cytokine secretion at the site of transection injury when compared with that in the hydrogel control group at 6-week post-injury. A conducive microenvironment for axonal regrowth and remyelination was generated by increasing expression levels of protein gene product 9.5 (PGP9.5) and myelin basic protein (MBP) in regenerating nerve tissues. PBA administration increased the relative gastrocnemius muscle weight percentage and maintained the intactness of muscle bundles when compared with those in the hydrogel control group.

**Conclusions:**

Suppressing the lengthened state of inflammation using PBA treatment favors axonal regrowth and remyelination following nerve transection injury. PBA treatment also regulates pro-inflammatory cytokine expression by inhibiting the transcriptional activation of NFκB-p65 and HDAC3 in SCs in vitro.

**Supplementary Information:**

The online version contains supplementary material available at 10.1186/s12974-021-02273-1.

## Background

Schwann cells (SCs) are resident cells of the peripheral nervous system (PNS) that are typically the first cell type to respond to injury. Unlike other tissues in which macrophages are the primary initiators of immune responses after injury, SCs in the PNS sense the damaging signals and upregulate the production of tumor necrosis factor-α (TNFα), interleukin-1α (IL-1α), and IL-1β [1]. The production of TNFα by SCs marks the beginning of an immune response at the injured site and regulates the recruitment of macrophages, with further strengthens the immune response to begin the process of Wallerian degeneration (WD) [2]. IL-1β influences mature SCs to undergo de-differentiation into repair SCs [3]. Together, SCs and macrophages clear myelin debris and create a conducive environment for successful axonal regeneration [4, 5].

Immune mediators, such as IL-1β, are only required during the WD process and are cleared prior to the initiation of nerve reinnervation, which implies that these factors play temporal roles during distinct stages of the process [6]. The balanced expression of TNFα has been shown to promote axonal regeneration, based on the observed effects of TNFα inhibition by the intraperitoneal and epineurial administration of a TNFα antagonist (etanercept) at the time of a crush injury [7, 8]. By contrast, chronic and prolonged inflammation caused by peripheral nerve injury can lead to complications, such as neuropathic pain [9]. Chronic inflammation can also obstruct nerve regeneration and functional recovery after PNS injury [10–12]. Therefore, unraveling the mechanisms that control the optimal level and duration of inflammatory mediators to support both the early phase of degeneration and the later phase of regeneration may identify possible therapeutic strategies for enhancing nerve regeneration.

Histone deacetylases (HDACs) are important epigenetic regulators that control gene expression through the post-translational modification of histone proteins. HDACs remove acetyl groups from N-acetyl lysine amino acids in histone proteins, causing chromatin compaction and transcriptional repression [13]. HDACs also function non-epigenetically via the modulation of transcription factors and associated proteins [14–17]. Each HDAC is activated in function-specific manner for different length of time. The upregulation of HDAC1/2 is important for the de-differentiation of myelinating SCs into repair SCs at earlier timepoints after lesion. HDAC1 and HDAC2 expression are also required to promote the redifferentiation of repair SCs when the remyelination process begins after injury, through the formation of a complex with the Zeb2 transcription factor and the suppression of the differentiation inhibitors Sox2 and Notch [18]. HDAC3 becomes downregulated 5-day post-transection injury in mice, indicating that it does not play a role in repair SCs, but HDAC3 expression is required during the final stages of nerve repair for the maintenance of SC myelination [19]. This evidence highlights the differential functions exhibited by various HDACs during different stages of repair and regeneration, which has been further supported by the findings from studies using HDAC inhibitors (HDACi). The inhibition of HDAC1/2 (by the HDAC1/2 inhibitor mocetinostat) for short periods during the early phase amplifies the conversion of myelinating SCs into the repair phenotype by upregulating the expression of c-Jun [20]. Similarly, the transient inhibition of HDAC3 (using HDAC3 inhibitors PDA106 or RGFP966) increases remyelination by epigenetically activating genes that encode proteins with promyelinating functions, such as phosphorylated protein kinase B (p-AKT), phosphorylated phosphoinositide 3-kinase (p-PI3K), and phosphorylated extracellular signal-regulated kinase (p-ERK) in SCs [21].

Sodium phenylbutyrate (PBA) is a non-competitive HDACi that selectively inhibits class I and IIa HDACs [22]. PBA exerts anti-inflammatory effects in lipopolysaccharide (LPS)-activated microglial cells by reducing the release of nitric oxide and the secretion of the cytokines TNFα and IL-6. Treatment with PBA has also been shown to reduce neuropathic pain induced by excessive TNFα production for 14 days in a chronic constriction injury rat model [23]. Although HDACis have commonly been used in disease models of neurodegenerative conditions to perform distinct neurotrophic and neuroprotective roles [24], the effects of HDAC modulation via PBA in chronic inflammation and the consequences of PBA administration on regeneration in the PNS have not yet been explicitly examined. In the current study, we explored the effects of PBA treatment on the maintenance of a timely and stable inflammatory reaction, both in vitro and in vivo. We also evaluated the mechanism of action through which PBA regulates pro-inflammatory cytokines in vitro. Finally, we investigated the beneficial outcomes associated with the regulated expression of inflammatory cytokines in SCs during axonal regeneration and remyelination following a peripheral nerve injury.

## Materials and methods

### RT4 SCs culture and treatment

We used RT4-D6P2T (RT4, ATCC number CRL-2768), a rat SC cell line, for the treatment with various inflammation inducers, such as LPS, TNFα, and M1-macrophage conditioned medium (M1-CM). Cells were maintained in Dulbecco’s modified Eagle medium (DMEM) supplemented with 10% heat-inactivated fetal bovine serum (FBS, HyClone, USA), 100 U/mL penicillin (Gibco, USA), and 100 µg/mL streptomycin (Gibco, USA) at 37 °C in a humidified incubator with 5% CO_2_. The cells were sub-cultured when they reached 90–100% confluency. For treatments, 3 × 10^5^ cells were seeded and grown for 20 h until they reached 80% confluency on a 6-cm culture dish. Various doses of LPS (0.1, 1, and 10 µg/ml) were used to test the inflammatory responses of SCs for 24 h. Similarly, cells at 80% confluency were treated with various doses of TNFα (0, 1, 5, and 10 ng/ml) for 24 h to confirm the initiation of inflammation. In addition, we used a human monocytic cell line (THP-1, Bioresource Collection and Research Center, Taiwan) to prepare M1-CM, according to a previously described protocol for M1 macrophage induction [25, 26]. The freshly collected M1-CM was used to treat RT4 SCs for 24 h.

HDACi PBA (sc-200652A, Santa Cruz) was reconstituted in filtered Milli-Q (MQ) water to prepare a stock solution (1 mM), which was stored at − 80 °C. A fresh aliquot was used for every experiment performed to obtain a working concentration of 150 µM PBA. It was used for either 1 or 24 h depending on the experiment.

### Enzyme linked immunosorbent assay (ELISA) for TNFα secretion

3 × 10^5^ cells were seeded in each well of a 6-well plate for 24 h prior treatment. After application of LPS or PBA for additional 24 h, the supernatant was collected and measured the TNFα release using the TNFα ELISA kit (438207, Biolegend, USA). Briefly, the cell supernatant with different treatments were added to the pre-coated wells and incubated at room temperature for 2 h with gentle shaking. Then, incubated with detection antibody for 1 h and the Avidin-HRP D for 30 min. The expression of blue color was developed after adding the Substrate Solution F (~ 2 min). The Stop solution was added to stop the reaction as soon as the color occured. The absorbance was measured immediately using ELISA reader (μQuant, Bio-Tek Instruments, Inc., USA) at 450 nm and 570 nm. The TNFα concentration was calculated after comparing the absorbance of each sample to the standard curve.

### Western blot

After the indicated treatment times, determined by the different experiments, the cells were collected for protein sample preparation. Briefly, the medium was discarded, and the cells were washed twice with ice-cold phosphate-buffered saline (PBS) and lysed using 200 µL radioimmunoprecipitation assay (RIPA) buffer (150 mM NaCl, 1 mM ethylene glycol tetraacetic acid [EGTA], 50 mM Tris, pH 7.4, 10% glycerol, 1% Triton X-100, 1% sodium deoxycholate, 0.1% sodium dodecyl sulfate [SDS], and protease inhibitor cocktail) [27]. Proteins were quantified using a Bradford assay kit (Thermo Fisher, USA), and 30 µg of proteins were separated by SDS–polyacrylamide gel electrophoresis (PAGE), followed by transfer onto a nitrocellulose membrane. The membrane was blocked with 5% milk and hybridized with various primary antibodies at 4 °C overnight. All antibodies were prepared in 1% milk at the following concentrations: poly-ADP ribose polymerase (PARP, 1:1000, 9542, Cell signaling, USA), TNFα (1:1000, ab1793, Abcam, USA), Toll-like receptor 4 (TLR4, 1:4000, ab203398, Abcam, USA), HDAC1 (1:5000, ab7028, Abcam, USA), HDAC2 (1:1000, ab32117, Abcam, USA), HDAC3 (1:5000, sc81600, Santa Cruz, USA), HDAC4 (1:1000, sc11418, Santa Cruz, USA), phospho-nuclear factor κB p65 (p-NFκB-p65, 1:1000, 3031, Cell signaling, USA), NFκB-p65 (1:1000, sc8008, Santa Cruz), and H3 (1:10,000, ab1791, Abcam, USA). Depending on the host species used to derive the primary antibodies, we used horseradish peroxidase (HRP)-conjugated anti-mouse (1:10,000, A9044, Sigma) and anti-rabbit (1:4000, AP132P, Millipore, USA) secondary antibodies to probe the membrane for 1–2 h at room temperature. The signal was detected on X-ray film using enhanced chemiluminescence (ECL) reagents (34096, Thermo Fisher, USA). The films were scanned and quantified the relative expression levels by ImageJ software (ImageJ, National Institutes of Health, USA).

### Nuclear and cytosolic fractionation for Western Blot and HDAC3 activity assay

To measure the target protein expression level in cell nucleus, the nuclear extraction kit (10009277, Cayman chemical, USA) was performed to isolate the cytosolic and nuclear fractions in according to the instruction manual. Briefly, the cells with various treatments were rinsed twice with ice-cold PBS and gently scraped off into the hypotonic buffer [28]. The obtained cells were lysed by adding 10% NP-40 reagent and immediately centrifuged at 13,200 rpm for 30 s. The supernatant was collected as cytosolic fraction. The pellet of nuclear fraction was resuspended in nuclear extraction buffer. The target protein expressions in cytosolic and nuclear fraction of each treatment were investigated by western blotting as aforementioned.

The fresh nuclear fractions were also used for measuring the HDAC3 activity using the fluorogenic HDAC3 assay kit (50073, BPS Bioscience, USA). Briefly, 0.15 μg of nuclear fraction protein for each sample were prepared in a total volume of 10 μl HDAC assay buffer. The solution of each treatment was then mixed with the master mix of HDAC substrate 3 (200 μM, 5 μl), BSA (1 mg/ml, 5 μl), and HDAC assay buffer (30 μl), transferred to the NUNC microtiter plate, and incubated at 37 °C for 30 min. The HDAC assay developer was added and incubated for 15 min. The samples were read in a fluorimeter (SpectraMax i3x Multi-Mode Microplate Reader, Molecular Devices, USA) at an excitation wavelength of 360 nm and emission wavelength of 450 nm. The relative expression level of HDAC3 activity after 24 h of different treatments were calculated after subtracted the blank sample reading value and compared to the reading value of control (non-treatment) group.

### Quantitative real time polymerase chain reaction (qRT-PCR) for measurement of gene expression

The mRNA expression for the inflammatory genes (TNFα, IL-1β, and IL-6) after LPS-, TNFα-induction and M1-CM treatment was assessed by qRT-PCR. In brief, after treatment with the inflammation inducers for specific times, the cells were gently washed twice with ice-cold PBS and 1 ml of TRIzol (Invitrogen, USA) was added to proceed for the RNA extraction [29]. The RNA concentration and quality were checked using a Nabi-UV/Vis Nano Spectrometer (MicroDigital Co., Ltd., Korea). The obtained RNA was reverse transcribed to complementary DNA (CDNA) using Oligo(dT) primers (Invitrogen, USA), dNTP Mix (Invitrogen, USA), 5 × First Strand Buffer (Invitrogen, USA), SuperScript III Reverse Transcriptase (Invitrogen, USA), RNaseOUT Recombinant Ribonuclease Inhibitor (Invitrogen, USA), and DTT (Invitrogen, USA), as per the manufacturer’s protocol (SuperScript III CellsDirect cDNA Synthesis System). The sequence of the primers used for qRT-PCR are listed in Table 1.

**Table 1:** The specific sequence of primers

Gene name	Forward (F)/ Reverse (R)	Primer sequence
TNFα	F	TCAACCTCCTCTCTGCCATC
	R	CCAAAGTAGACCTGCCCAGA
IL-1β	F	CTGTCCTGCGTGTTGAAAGA
	R	CTGCTTGAGAGGTGCTGATG
IL-6	F	AGGAGACTTGCCTGGTGAAA
	R	CAGGGGTGGTTATTGCATCT
GAPDH	F	CATCAAGAAGGTGGTGAAGC
	R	TGACAAAGTGGTCGTTGAGG

### Immunofluorescence staining of cell

Coverslips coated with poly-l-lysine (P6282, Sigma) were used for the seeding of SCs. Immunofluorescent staining was performed to visualize the protein expression and distribution after treatment for 0.5, 1, 3, and 6 h [29, 30]. Briefly, the medium was discarded, cells were gently washed twice with ice-cold PBS, followed by fixation with 4% paraformaldehyde (P6148, Sigma) for 10 min. Triton X-100 (0.1%) was used to permeabilize the cells, and 5% bovine serum albumin was used for 1 h to block non-specific binding at room temperature. The primary antibody against NFκB-p65 (1:500) was applied overnight at 4 °C. A secondary antibody conjugated to Alexa Flour (A11003, Invitrogen) and 4′,6-diamidino-2-phenylindole (DAPI) were used to visualize the primary antibody and the nuclei, respectively, followed by the mounting of coverslips onto slides. Five images were captured from five random visual fields within each coverslip, using a 40 × objective lens on a spinning disk confocal microscope (DSU, Olympus). The images were acquired and quantified using ImageJ.

### Establishment of sciatic nerve transection and conduit model for nerve regeneration

To perform the sciatic nerve transection injury, we used 8-week-old male Sprague Dawley (SD) rats (300–350 weight, BioLASCO Taiwan Co. Ltd., Taipei, Taiwan), which were maintained at the Animal Center of National Cheng Kung University (NCKU). The experimental procedures were reviewed and approved by the Institutional Animal Care and Use Committee (IACUC-105224) at NCKU. We performed all experiments in accordance with the relevant IACUC and ARRIVE guidelines. The experiment included three groups: sham (negative control), hydrogel (positive control, no treatment) and PBA (treatment with 150 µM PBA). A total of 15 animals were randomly divided into 3 groups (5 animals per group). The ‘*N* = 4’ number mentioned in the figures refer to the total number of animals used for final sample harvesting in each group to provide image data and analysis. Only few rats passed out during surgery or failure in sample harvesting. The rats were anesthetized by an intraperitoneal injection of 25 mg/kg Tiletamine and 25 mg/kg Zolazepam (Zoletil, Virbac, France). Since the gap > 1 cm in rat sciatic nerve is considered as a critical gap that usually does not spontaneously regenerate [31, 32], the nerve transection and conduit surgical procedure was adapted from our previously study [27, 33] with the gap of 1.2 cm to test the promotion of nerve regeneration in current study. Briefly, the experiments were performed on the left sciatic nerve of the rats and the right limbs were untouched throughout the surgery. For the animals in the sham group, the nerve was exposed but no nerve transection occurred. In the hydrogel and PBA groups, nerve transection was performed 1 cm distal to the sciatic notch. Proximal and distal stumps were inserted 0.15 cm into a 1.5 cm long silicone tube conduit and sutured using No. 9–0 nylon to create a 1.2 cm nerve gap between two transected nerve ends. The hydrogel control group received 100 µl of crosslinked hydrogel (HyStem cell culture scaffold kit, Sigma HYS020, USA) to test the regeneration ability without drug treatment (only hydrogel effect). The PBA group received 150 µM of PBA mixed in 100 µl of crosslinked hydrogel that was injected into the silicon conduit. After 6-week post-injury, the rats in each independent set of experiments were randomly euthanized with carbon dioxide by one investigator, the second investigator harvested the gastrocnemius muscle and regrown nerve within the silicon conduit, and the third investigator immediately weighed the muscles and removed the fascia from the nerves to fix the muscles and nerves using 4% paraformaldehyde for 24 h. The harvested tissues were used for further histological assessments to analyze nerve inflammation, regeneration, and myelination.

### Immunohistochemistry (IHC)

The fixed nerves were then dehydrated using an ethanol gradient manner, ranging from 100 to 70% ethanol, for 1 h each, followed by clearance using xylene (5 times, 30 min each) [27, 34]. Afterward, the tissue was immersed in five changes of paraffin (30 min each) at 65 °C to clear the xylene from the tissue. Eventually, the tissue was embedded in a paraffin block, and 10 µm transverse sections were cut and transferred to amino propyltriethoxy silane coated slides (APS, Matsunami Glass Ind., ltd., Japan). Each slide had five sections of the middle section of the nerve. The slides were stored at room temperature until staining. For staining, the sections were rehydrated in xylene and ethanol, followed by antigen retrieval using antigen retrieval buffer (Thermo Fisher, USA). The slides were blocked using the blocking buffer provided with the immunohistochemistry (IHC) kit (ab232466, Abcam, USA). Afterwards, we used specific primary antibodies against PGP9.5 (1:100, ab109261, Abcam, USA), S100β (1:1000, ab52642, Abcam, USA), myelin basic protein (MBP, 1:500, 78896 T, Cell signaling), TNF-α (1:1000, ab1793, Abcam, USA), IL-1β (1:1000, ab9722, Abcam, USA), and IL-6 (1:1000, ab9324, Abcam, USA), by overnight incubation at 4 °C. Next, we used an HRP-conjugated secondary antibody for 10 min, followed by detection using 3,3′-diaminobenzidine (DAB) reagent for 40 s–2 min. The sections were then stained with hematoxylin and mounted. Images were obtained at 40 × magnification using Magnafire software with an Olympus microscope.

### Myelin sheath staining by Luxol fast blue solution

The transverse sections of paraffin embedded nerve tissue were used to perform myelin staining with a Luxol fast blue stain kit (ab150675, Abcam, USA) [27]. Briefly, nerve sections were deparaffinized and rehydrated in distilled water. The slides were incubated at room temperature in Luxol fast blue solution for 14–16 h, rinsed with distilled water, and dipped in lithium carbonate solution for 20 s. Until visible myelin sheath structure was obtained (2.5 min), the additional background color was removed by repeated dipping in 70% alcohol solution. After rinsing in water and dehydration in 100% alcohol, the slides were examined under a 40 × objective lens with the microscope, and images were obtained.

### Quantification of images and their analysis

Western blot, immunofluorescence staining, and IHC images were analyzed using image analysis software program (ImageJ, National Institutes of Health, USA). Densitometric analysis was measured to quantify the protein expression in each western blot by determining the band intensities on the film [35]. Relative expression levels of target proteins were normalized to the internal control β-actin (loading control) and the expression of target protein under control condition (without LPS or PBA). For the IHC image quantification, we referred to the protocol described by Alexandra et al. [36]. In brief, a threshold value was set to remove the background signal after the deconvolution of IHC images, followed by the quantification of the DAB signal within the image. Next, we measured the average size of the nucleus. We also quantified the hematoxylin signal using a similar process as was used for the DAB signal. Then, we normalized both DAB and hematoxylin intensity against the nuclear intensity. The analysis was performed by acquiring three images from three random visual fields in three different sections for each nerve tissues harvested from different treatments. The average intensity of DAB signal in IHC images was calculated and the mean value of 3 visual fields was chose to represent the target protein expression.

### Statistical analysis

The figure legends indicate the total numbers of biological replicates (*n*) used for each experiment for analysis. All experiments were performed on separate days, following the same protocols. We used GraphPad Prism to perform statistical analyses. The tests used to analyze the in vivo and in vitro experimental results were one-way analysis of variance (ANOVA) and paired two-tailed Student’s *t* test, respectively. The results were considered significant for *P* value < 0.05.

## Results

### LPS treatment induces inflammation by increasing TLR4, HDAC2/3/4, TNFα and transient NFκB-p65 activation in RT4 SCs

**Fig. 1:**
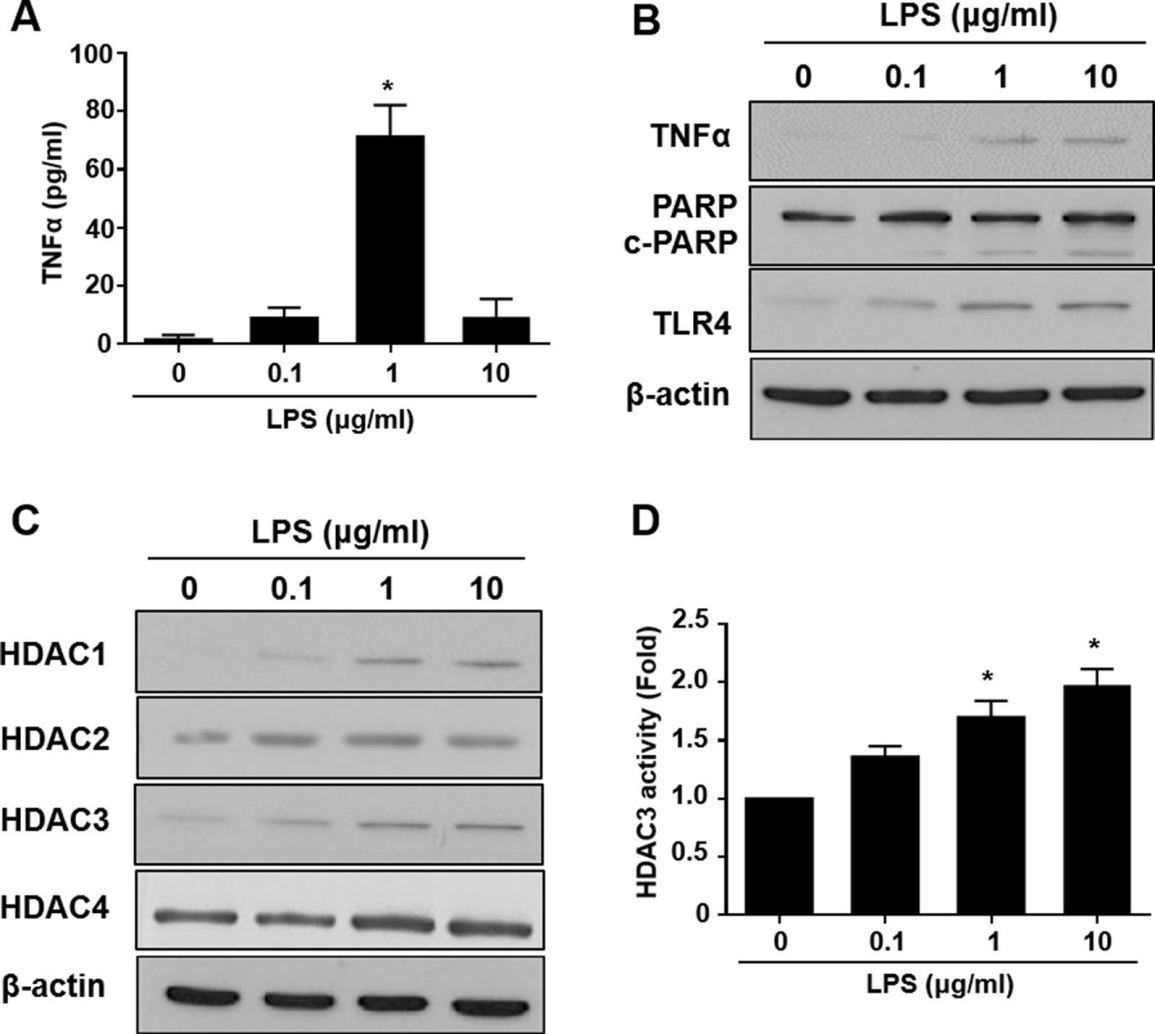
Inflammatory response and the modulation of HDAC expression by LPS in RT4 SCs. **A** Secretion of TNFα after LPS induction for 24 h were measured by ELISA. **B** Representative western blot data of changes in the expression of TNFα, c-PARP, and TLR4 expression in a dose-dependent manner, following LPS induction for 24 h. **C** Expression profile of HDAC1, 2, 3, and 4 after LPS induction for 24 h. **D** LPS-induced increases of HDAC3 activity were confirmed by HDAC3 activities assay. *n* = 4. Significance was assessed by one-way ANOVA. The data are presented as the mean ± SEM. *p < 0.05 versus 0 μg/ml LPS. *LPS* lipopolysaccharide, *SCs* Schwann cells, *TNFα* tumor necrosis factor α, *c-PARP* cleaved poly (ADP) ribose polymerase, *HDAC* histone deacetylase, *TLR4* toll-like receptor 4, *ANOVA* analysis of variance, *SEM* standard error of the mean

The optimal dose of LPS required to induce inflammation in RT4 SCs was verified by examining the secretory inflammatory cytokine (TNFα, Fig. 1A), and protein expression level of TNFα and cell apoptosis, based on the level of cleaved PARP (c-PARP, Fig. 1B). TNFα secretion and expression increased after stimulating RT4 SCs with 1 µg/ml of LPS for 24 h, but not after treatment with 10 µg/ml of LPS for 24 h (Fig. 1A, B). Both 1 and 10 µg/ml LPS treatment for 24 h significantly induced PARP cleavage in RT4 SCs (quantification in Additional file 1: Fig. S1A). A relatively high level of c-PARP expression was induced in response to 10 µg/ml LPS treatment, suggesting that this treatment level represented an overdosage that could not be rescued in later experiments. The LPS also increased the gene expression of inflammatory cytokines IL-1β, but not the IL-6 (Additional file 2: Fig. S2A). Toll-like receptor (TLR) signaling is critical during the initiation of inflammation, and SCs are known to express TLR4 [37]. We speculated that the binding of LPS molecules might activate TLR4 in RT4 SCs. A significant increase in TLR4 expression was observed after treatment with either 1 or 10 µg/ml LPS for 24 h (Fig. 1B).

In microglia, LPS-induced inflammation through TLR4 activation is known to result in HDAC activation [38]. The ability of LPS-induced inflammation to modulate HDAC expression in RT4 SCs was investigated by measuring the expression levels of HDAC1, HDAC2, HDAC3, and HDAC4 (Fig. 1C). Treatment with 1 or 10 μg/ml LPS significantly increased the expression levels of HDAC2, HDAC3, and HDAC4 but not HDAC1 (quantification in Additional file 1: Fig. S1B). The induction of inflammatory responses in RT4 SCs was confirmed by treatment with other inflammation inducers, including TNFα (Additional file 2: Fig. S2B) and M1-CM (Additional file 2: Fig. S2C), which increased inflammatory gene expression (TNFα, IL-1β, and IL-6). Taken together, these results suggested that treatment with 1 μg/ml LPS was sufficient to induce inflammation in RT4 SCs. The induction of inflammatory responses in SCs by various inducers occurs through common increases in TNFα, TLR4, HDAC2 and 3, and PARP cleavage.

Because NFκB serves as an intermediary during the inflammatory response, activating the transcription of inflammatory cytokines [39], we further tested the activation of NFκB-p65 signaling at various timepoints (0.5, 1, 3, 6, 12, and 24 h) after LPS treatment (1 μg/ml).

**Fig. 2:**
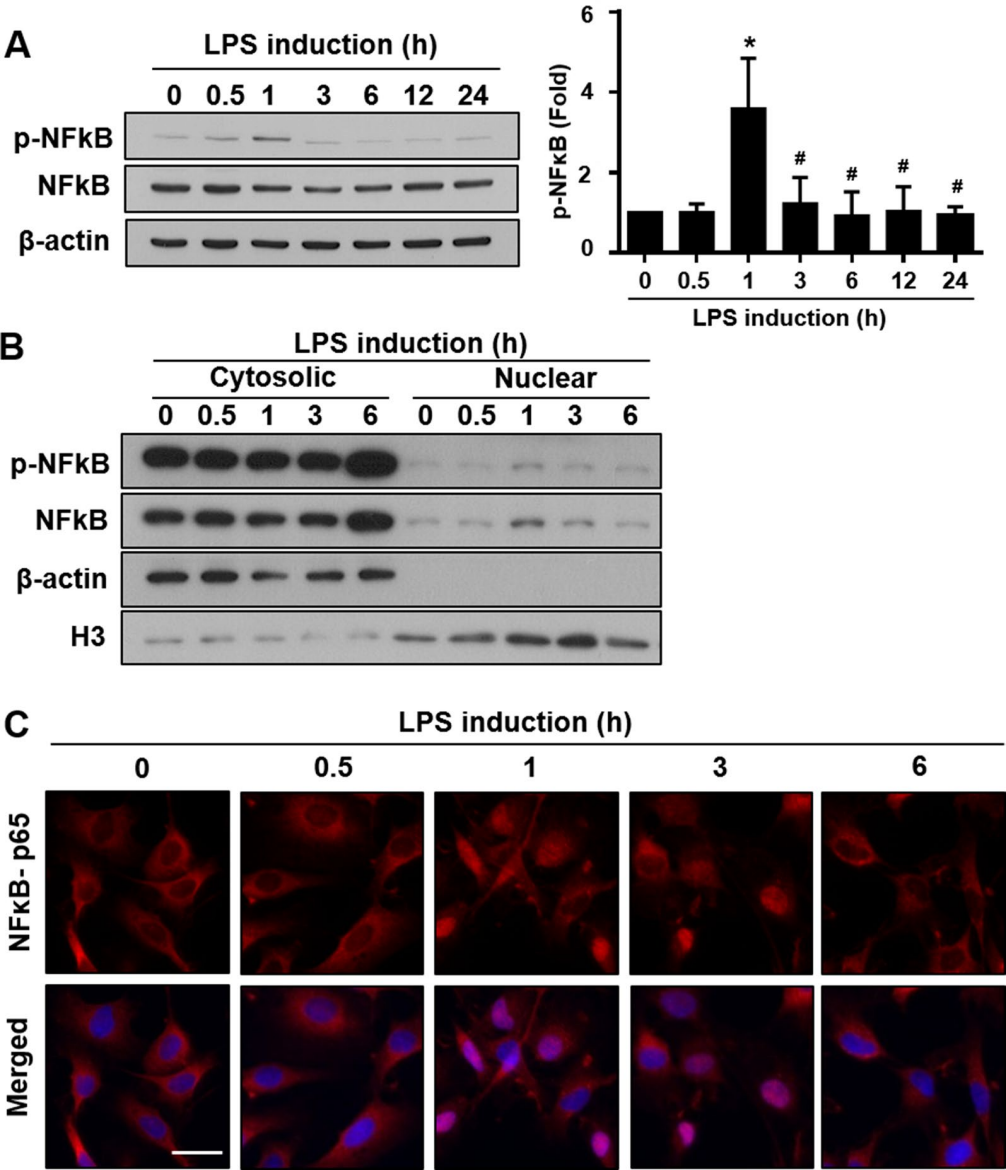
LPS induces the phosphorylation and translocation of NFkB-p65 at 1 h after LPS treatment. **A** Western blot data and quantification showed the phosphorylation and expression pattern of NFκB-p65 at various timepoints after LPS induction. **B** Time course of p-NFkB-p65 expressions in cytoplasm and nuclear fraction confirmed the translocation of NFkB-p65 after LPS stimuli. **C** Immunofluorescence staining for NFκB-p65 showing the start of NFκB-p65 nuclear translocation from 1 to 3 h, followed by a decline at 6 h. *n* = 4. *LPS* lipopolysaccharide, *p-NFκB-p65* phospho-nuclear factor κB p65 subunit

The LPS may bind to the TLR4 to induce activation and translocation of NFκB into the nucleus [40]. The phosphorylation of NFκB-p65 indicated that NFκB-p65 activation occurred 1 h after LPS treatment (Fig. 2A). The LPS induced the translocation of phosphorylated NFκB-p65 into the cell nucleus was confirmed by cytosolic and nuclear protein fractions (Fig. 2B). The transient translocation of NFκB-p65 into the nucleus was also observed by immunofluorescent staining after 1 h of LPS treatment in RT4 SCs (Fig. 2C). The time course of HDAC3 expressions following LPS induction was measured as shown in Additional file 3: Fig. S3A. These data further suggested that the transient translocation and activation of the transcription factor NFκB-p65 might induce these pro-inflammatory markers during LPS-induced inflammation.

### PBA inhibits transient NFκB-p65 activation and TLR4 to reduce SCs inflammation through the suppression of HDAC3 expression

**Fig. 3:**
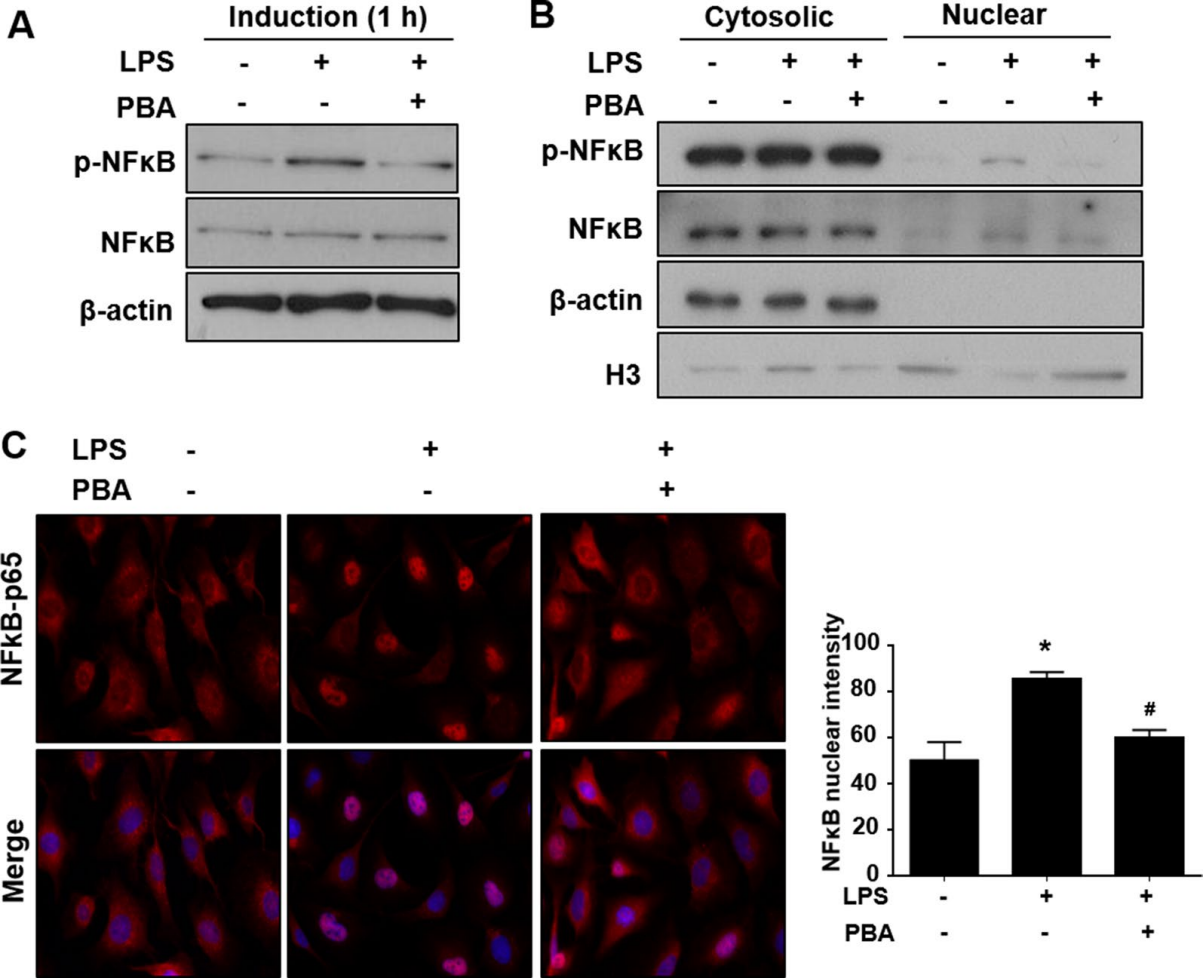
PBA prevents the LPS-induced NFκB-p65 phosphorylation and translocation to the nucleus. **A** Western blot analysis and quantification showing a significant decrease in phosphorylation of NFκB-p65 following combined PBA and LPS treatment. *n* = 4. **B** Reduction of activated NFκB-p65 into the nucleus was revealed after PBA treatment. **C** Immunofluorescence staining shows the reduced nuclear translocation of NFκB-p65 after induction with LPS and PBA together and its representative graph. *n* = 4. Significance was assessed by one-way ANOVA. Data are presented as the mean ± SEM. *p < 0.05 versus no LPS and no PBA. ^#^*p* < 0.05 versus only LPS. *PBA* sodium phenylbutyrate, *LPS* lipopolysaccharide, *NFκB-p65* nuclear factor κB p65 subunit, *ANOVA* analysis of variance, *SEM* standard error of the mean

We further investigated the effects of PBA on the LPS-induced phosphorylation and nuclear localization of NFκB-p65. Because our earlier results demonstrated that the phosphorylation of NFκB-p65 and nuclear localization occurred 1 h following 1 μg/ml LPS treatment, RT4 SCs were treated with 150 μM PBA and 1 μg/ml LPS combined for 1 h. The treatment with PBA decreased the LPS-induced phosphorylation of NFκB-p65 (Fig. 3A). The translocation of NFκB into the nucleus was also significantly inhibited in RT4 SCs treated with both PBA and LPS (Fig. 3B, C). These data indicated that PBA has the potential to prevent LPS-induced NFκB-p65 pathway activation, further regulating pro-inflammatory cytokine expression.

**Fig. 4:**
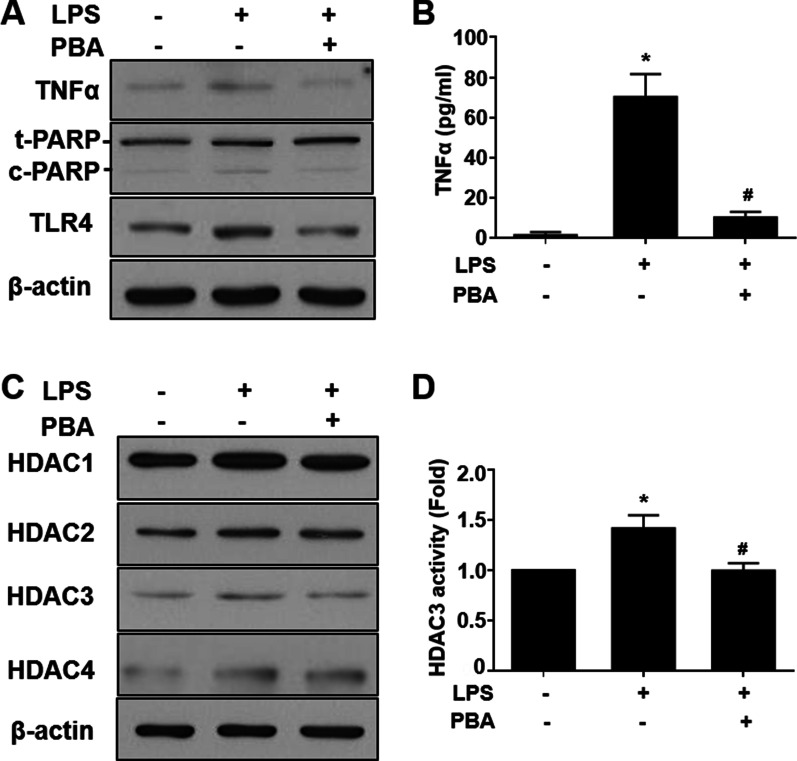
PBA reverses LPS-induced inflammatory effects in RT4 SCs. **A** Western blot analysis shows the decreased expression levels of TNFα, c-PARP, and TLR4 following LPS and PBA co-treatment for 24 h. **B** LPS-induced secretion of TNFα was inhibited by PBA treatments. **C** Western blot depicting the changes in HDAC3 expression levels but not in the levels of the other HDACs after 24 h of treatment with LPS and PBA. **D** HDAC3 activity induced by 24 h of treatment with LPS was also suppressed after PBA treatment. *n* = 4. Significance was assessed by one-way ANOVA. Data are presented as the mean ± SEM. *p < 0.05 versus no LPS and no PBA. ^#^*p* < 0.05 versus only LPS. *LPS* lipopolysaccharide, *PBA* sodium phenylbutyrate, *SCs* Schwann cells, *TNFα* tumor necrosis factor α, *c-PARP* cleaved poly (ADP) ribose polymerase, *TLR4* toll-like receptor 4, *HDAC* histone deacetylase, *ANOVA* analysis of variance, *SEM* standard error of the mean

The long-term effects of PBA on reduced inflammatory responses were confirmed by investigating the expression levels of LPS-induced downstream targets, such as c-PARP and TNFα. The anti-inflammatory and anti-apoptotic effects of PBA were validated by incubating RT4 SCs with PBA (150 µM) and LPS (1 µg/ml) together for 24 h (Fig. 4A, quantification in Additional file 4: Fig. S4A). PBA attenuated the secretion of the LPS-induced pro-inflammatory cytokine TNFα (Fig. 4B). A similar significant reduction in the expression level of c-PARP was observed with PBA treatment (Additional file 4: Fig. S4A). Because we previously identified that LPS triggered the RT4 SC inflammatory response through the activation of TLR4, we also verified the potential of PBA in preventing TLR4 activation. Among all activated HDACs, PBA was able to suppress the enhanced expression of HDAC3 in response to LPS treatment for 24 h (Fig. 4C). However, no significant changes were observed in the expression levels of HDAC1, 2, or 4 after 24 h of the co-treatment of RT4 SCs with both LPS and PBA compared with the levels observed for LPS treatment alone (quantification in Additional file 4: Fig. S4B). Inhibition of LPS-induced HDAC3 activation was confirmed by measuring the HDAC3 activities after 24 h of PBA treatment (Fig. 4D). These data indicated that PBA attenuated the secretion of the pro-inflammatory cytokine TNFα through the downregulation of HDAC3 protein expression.

### Administration of PBA reduces the prolonged secretion of inflammatory cytokines at a peripheral nerve injury site

**Fig. 5:**
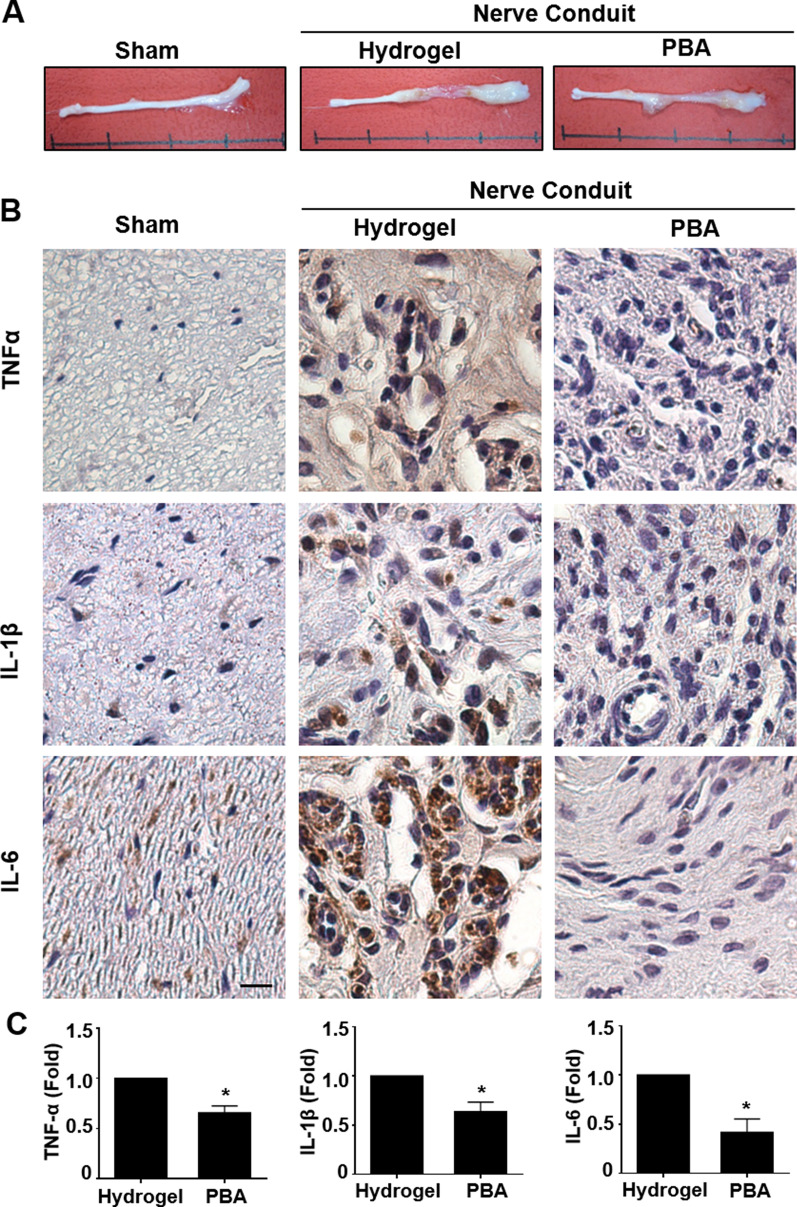
PBA administration reduces chronic inflammation after peripheral nerve injury. **A** Representative pictures of the harvested sciatic nerves after 6 weeks from three groups of rats. **B** Representative images of immunohistochemistry staining against TNFα, IL-1β, and IL-6 in paraffin-embedded sections obtained from the middle region of the nerves in each treatment group and their and their respective quantification for the expression. *n* = 4 different rats for each group. Scale bar = 20 μm. Significance was assessed by Student’s *t* test. *p < 0.05 versus hydrogel control group. *PBA* sodium phenylbutyrate, *TNFα* tumor necrosis factor α, *IL* interleukin

Pro-inflammatory responses in the conduit of nerve segment were induced after 6-week post-injury in the nerve transection and silicone conduit sutured rats [26, 33]. We observed that PBA had a beneficial effect on the inhibition of pro-inflammatory cytokines in vitro; therefore, we investigated the therapeutic potential of PBA for the modulation of inflammation in the nerve conduit after sciatic nerve transection. Because 150 µM PBA was an effective dose in our cell experiments, we delivered 150 µM PBA with mixture of hydrogel through the conduit that was used to connect the two nerve stumps in a relative isolated microenvironment without dilution of drug dosage. The nerves were harvested 6-week post-injury, and better gross morphology was observed in the PBA group than in the hydrogel control group (Fig. 5A). We assessed the nerve region within the conduit for the expression levels of TNFα, IL-1β, and IL-6 using IHC after 6 weeks of nerve regeneration (Fig. 5B) [41–44]. The IHC staining revealed a significant decline in TNFα, IL-1β, and IL-6 expression levels in the nerve sections from the PBA group compared with the hydrogel control group (Fig. 5C). These results demonstrated the anti-inflammatory effects exerted by PBA, resulting in the reduced long-term expression of inflammatory cytokines up to 6-week post-injury.

### PBA enhances axonal regrowth and remyelination to promote the peripheral nerve regeneration

**Fig. 6:**
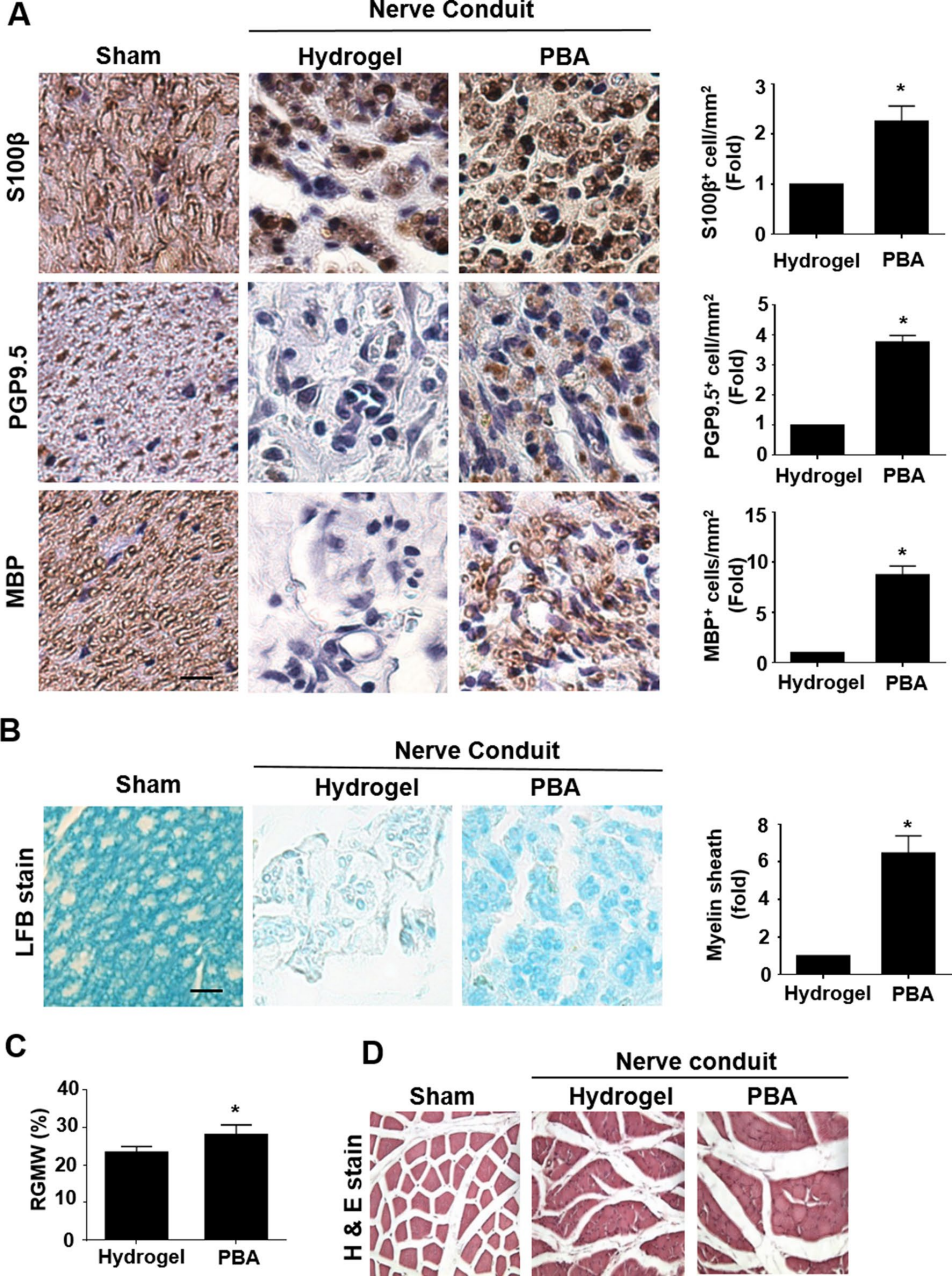
PBA administration enhances axonal regeneration and myelination and prevents muscle atrophy after 6 weeks of sciatic nerve transection injury. **A** Representative images of immunohistochemistry staining for S100β, PGP9.5, and MBP in the middle region of the regenerating nerve from three groups with quantification of positive-stained cells. **B** Myelin sheath staining using Luxol fast blue in the middle part of the nerve and quantification of positive-stained myelin sheath. **C** Graph representing the comparison of RGMW between the hydrogel control and PBA groups. **D** H&E staining of sections of the left gastrocnemius muscle in three groups. *n* = 4 different rats for each group. Scale bar = 20 μm. Significance in (**C**) was assessed by Student’s *t* test. *p < 0.05 versus hydrogel control group. *PBA* sodium phenylbutyrate, *PGP9.5* protein gene product 9.5, *MBP* myelin basic protein, *LFB* luxol fast blue, *RGMW* relative gastrocnemius muscle weight

The sustained inflammatory environment hinders the regenerative capacity of peripheral nerves; therefore, we evaluated the outcome of PBA administration on the effects of nerve regeneration [45]. The regenerative cues were assessed by IHC staining for several markers associated with SCs, axonal regeneration, and remyelination. A marked increase in SC number (approximately twofold) was observed based on S100β staining (Fig. 6A). The regrowth of axons (3.5-fold) was also visualized using the positive staining of the axonal marker PGP9.5 (Fig. 6A). In addition, the myelination of SCs was promoted, as determined by an increase in MBP staining (Fig. 6A). We also used Luxol fast blue staining to examine the remyelination status of regrown axons after 6 weeks and found out that the PBA group had an increase in the number of cells with intact myelin sheath structure compared with that in the hydrogel control group (Fig. 6B).

The conductive microenvironment was investigated by studying the level of nerve innervation to the targeted gastrocnemius muscle as an indicator of the successful regeneration outcome [26, 33]. The degree of nerve reinnervation was analyzed by determining the relative gastrocnemius muscle weight (RGMW) and performing a histological examination using cross-sectional image of innervated muscle tissues. RGMW was calculated as the ratio of the muscle weight from the left (transected) side to that on the right (untransected) side. The PBA treatment group showed a significant increase in RGMW compared with the hydrogel control group (Fig. 6C). Improved nerve reinnervation was further confirmed by the histological images of muscle tissue, which demonstrated thicker and more intact muscle fiber bundles in the PBA group than in the hydrogel control group (Fig. 6D). Taken together, these results indicated the therapeutic potential of PBA for promoting successful nerve regeneration by enhancing axonal regrowth, SC myelination, and the reinnervation of the targeted muscle tissue.

## Discussion

An acute inflammatory response following injury is considered to be beneficial for regenerative outcomes [46, 47]. Similarly, in response to a peripheral nerve injury, WD induces the expression of pro-inflammatory cytokines, such as increased levels of TNFα and interferon-γ (IFNγ) [48]. Typically, this inflammatory response becomes dampened 2–3 weeks after injury, followed by the elimination of macrophages [49, 50]. However, a prolonged inflammatory response, correlated with an altered cytokine expression profile, exacerbates nerve regeneration following peripheral nerve injury [11]. Therefore, an optimal immune response associated with the careful orchestration of temporal and spatial cytokine and chemokine expression is necessary to achieve successful nerve regeneration. Studies based on the sciatic nerve axotomy model have demonstrated that acute peripheral nerve injury can trigger an immunosuppressive milieu prior to a decline in inflammatory gene expression through the stimulated expression of negative regulators of the immune response and the generation of M2-phenotypic macrophages, which are anti-inflammatory in nature, to control the damaging effects caused by an exaggerated immune response [51]. In support of this study, another group showed an enhanced rate of axonal regeneration in response to the inhibition of the inflammatory cytokine TNFα immediately following a sciatic nerve crush injury [7, 52]. Kukkar et al. report the anti-nociceptive effects of PBA administration during chronic compression injury and the regulatory effects on the expression of the pro-inflammatory cytokine TNFα [23]. These studies reflect the importance of a regulated immune response for the promotion of wound repair and the maintenance of tissue homeostasis [53]. The beneficial results of PBA application under inflammatory conditions, such as in rat models of permanent ischemic stroke, osteoarthritis, or acute lung injury models, were shown to decrease the release of pro-inflammatory mediators IL-1β, TNFα, and IL-6 [54–56]. The prolong presence of Iba1-positive macrophages and activation of pro-inflammatory cytokine were found to delay of nerve regeneration in *Cryab*^*−/−*^ mice [57]. The sustained high expression of pro-inflammatory M1 macrophages and decrease of anti-inflammatory M2 macrophages hindered the regeneration. The continued elevation of inflammatory cytokine expression was observed in the rats that received hydrogel in the silicone conduit after 6-week post-injury and resulted in the failure of nerve regeneration across the critical nerve gap [26]. Here, the administration of PBA attenuated the prolonged inflammation following the sciatic nerve transection injury, as demonstrated by our in vivo results.

Epigenetic mechanisms, especially histone modifications, are known to play roles in inflammatory pathways and various inflammation-associated disorders, such as obesity, cancer, and diabetes [58]. However, the regulatory mechanisms that induce epigenetic modifiers to balance the expression of inflammatory mediators during peripheral nerve regeneration have not yet been delineated. The present study provides the first evidence regarding the potential of PBA to suppress persistent inflammation following nerve injury. In addition, during in vitro experiments, PBA showed anti-inflammatory effects and suppression of HDAC3 activity and protein expression via the NFκB-p65 pathway. The anti-inflammatory outcomes described in response to other HDAC inhibitors indicate the potential role played by HDACs in the transcriptional regulation of pro-inflammatory cytokines [59–62]. Therefore, the reduced expression levels of pro-inflammatory cytokines observed in our study may be attributable to the inhibition of HDAC3 activity and reduction in activation of NFκB-p65 by PBA. The HDACi valproic acid (VPA), when implanted in a conduit following sciatic nerve injury in rats, was found to be beneficial for improving nerve regeneration and functional recovery [63, 64]. In the present study, PBA administration rescued the nerves from the persistent elevation of pro-inflammatory cytokine levels, which accelerated axonal regeneration and remyelination. Our studies are in concert with the results reported by other research groups, in which the attenuation of TNFα upregulation and reduced apoptosis following sciatic nerve crush injury accelerated nerve regeneration following natto treatment [65]. In addition, TLR4-deficient mice showed the reduced activation of SCs after sciatic nerve lesions, slowing the initiation of WD and the process of demyelination, as indicated by the presence of persistent myelin debris [66, 67]. Additional future studies remain necessary to better understand the impacts of PBA on the early inflammatory phase during WD in our animal model. However, a potentially direct anti-inflammatory effect of PBA must also be considered.

Using LPS-stimulated RT4 SCs, we demonstrated that PBA inhibited the LPS-mediated induction of TLR4 signaling and blocked the activation of NFκB-p65 and HDAC3, dampening the inflammatory response through the attenuated production of inflammatory mediators, such as TNFα. Similarly, the use of rifampin to inhibit TLR4 activation suppresses NFκB-p65 and reduces the overproduction of inflammatory cytokines in mouse microglial cells [68]. The findings reported by other groups have also indicated the importance of HDAC3 for the regulation of inflammatory cytokine expression. The effects of HDAC3-specific inhibition on reduced NFκB-p65 transcriptional activity and the reduced expression levels of pro-inflammatory cytokines have been demonstrated in LPS-treated RAW 264.7 macrophage cultures and pulmonary inflammation model systems [69, 70]. Our work revealed that the inhibitory spectrum of PBA varied across class I and IIa HDACs. PBA potently suppresses the protein expression and activity of HDAC3, in contrast to no or modest effects on other class I HDACs (HDAC1 and 2) and class IIa HDACs (HDAC4). A previous study also reported the distinct inhibitory effects of PBA on different HDACs, showing the strong suppression of HDAC2 among class I HDACs and no suppression of class IIa HDACs in cardiomyocytes, suggesting the selectivity of PBA toward class I HDAC inhibition [71]. HDAC inhibitors act through various mechanisms, such as the inhibition of HDAC protein expression, disrupting HDAC nuclear translocation, or suppressing HDAC transcriptional activity [72]. Further investigation into how PBA affects the interplay of NFκB-p65 nuclear translocation and HDAC3 activities would provide valuable molecular mechanism to mediate inflammation. The modulation of HDACs and histone 3 methylation at lysine 9 (H3K9) using the HDAC activator ITSA-1 was shown to be beneficial for the prevention of venous endothelial cell peel-off during vein-graft diseases and failures [29]. Therefore, in addition to examining HDAC expression, epigenetic regulations mediated by methylation should also be explored for their potential hindrance of peripheral nerve regeneration caused by extensive inflammation.

## Conclusions

In brief, we demonstrated the effectiveness of PBA in the regulation of pro-inflammatory cytokine expression (TNFα, IL-1β, and IL-6) in SCs. The reduced expression of pro-inflammatory cytokines corresponded with the suppression of HDAC3 protein levels and the hindered nuclear translocation of NFκB-p65. Although we demonstrated that the inhibition of both NFκB-p65 and HDAC3 expression reduced inflammatory responses, additional studies remain necessary to validate the influence of PBA on the transcriptional activity of NFκB-p65 and HDAC3 and their interaction. However, this study sheds light on the potential of PBA to regulate the balance of pro-inflammatory cytokine expression, resulting in improved axonal regeneration, remyelination, and reinnervation.

## Supplementary Information

**Additional file 1: Figure S1.** LPS-induced inflammation and the activation of TLR4 affect the Class I and Class IIa HDAC expression profile. (A) Quantification of the western blot data from Fig. 1. (B) for the expression levels of TNFα, c-PARP, and TLR4 showed an increase in dose-dependent manner after 24 h treatment with LPS. (B) Quantification of western bot data from Fig. 1 (C) shows a change in the expression pattern of HDACs. *n* = 4. Significance was assessed by one-way ANOVA. The data are presented as the mean ± SEM. *p < 0.05 versus 0 μg/ml LPS induction. LPS: lipopolysaccharide; TNFα: tumor necrosis factor α; c-PARP: cleaved poly (ADP) ribose polymerase; TLR4: Toll-like receptor 4; HDAC: histone deacetylase; ANOVA: analysis of variance; SEM: standard error of the mean.

**Additional file 2: Figure S2.** The mRNA expression of inflammatory cytokines in RT4 SCs in response to various inflammation inducers. (A) qRT-PCR results show changes in the mRNA expression profile of TNFα, IL-1β, and IL-6, in a time-course manner after induction with 1 μg/ml LPS. (B) Increase in levels of inflammatory cytokines in a dose-dependent manner after induction of cells with TNFα for 24 h. (C) Treatment with M1-CM for 24 h significantly increased the mRNA expression levels of TNFα, IL-1β, and IL-6. *n* = 4. Significance was assessed by one-way ANOVA. The data are presented as the mean ± SEM. *p < 0.05 versus 0 h of LPS treatment in (A). *p < 0.05 versus 0 μg/ml TNFα in (B). *p < 0.05 versus 0 h M1-CM treatment in (C). SCs: Schwann cells; LPS: lipopolysaccharide; TNFα: tumor necrosis factor α; qRT-PCR: quantitative real-time polymerase chain reaction; IL: interleukin; M1-CM, macrophage M1 conditioned media; ANOVA: analysis of variance: SEM: standard error of the mean.

**Additional file 3: Figure S3.** LPS induces HDAC3 activation after 24 h. (A) Western blot analysis and quantification showing the expression time for HDAC3 after LPS induction in RT4 SCs. *n* = 4. Significance was assessed by one-way ANOVA. The data are presented as the mean ± SEM. *p < 0.05 versus 0 h LPS induction. #*p* < 0.05 versus 1 h of LPS treatment. LPS: lipopolysaccharide; HDAC: histone deacetylase; SC: Schwann cell; ANOVA: analysis of variance; SEM: standard error of the mean.

**Additional file 4: Figure S4.** LPS-induced inflammation, the activation of TLR4, and HDAC modulation are altered by PBA treatment in RT4 SCs. (A) Quantification of western blot data from Fig. 4 (A) for the expression levels of TNFα, c-PARP, and TLR4 revealed a decrease in their expression following LPS and PBA co-treatment for 24 h. (B) Quantification of western blot data from Fig. 4 (C) shows the reduced expression of HDAC3 with LPS and PBA co-treatment. *n* = 4. Significance was assessed by one-way ANOVA. The data are presented as the mean ± SEM. *p < 0.05 versus no LPS and PBA. ^*#*^*p* < 0.05 versus only LPS. PBA: sodium phenylbutyrate; LPS: lipopolysaccharide; SCs: Schwann cells; TNFα: tumor necrosis factor α; c-PARP: cleaved poly (ADP) ribose polymerase; TLR4: Toll-like receptor 4; HDAC: histone deacetylase; ANOVA: analysis of variance: SEM: standard error of the mean.

## Data Availability

The data obtained from this study are included in the article or the supplementary files.

## Abbreviations

ANOVA: Analysis of variance
DAB: Diaminobenzidine
DMEM: Dulbecco’s modified Eagle medium
FBS: Fetal bovine serum
HDAC: Histone deacetylase
HDACi: HDAC inhibitor
HRP: Horseradish peroxidase
H: Hour
IHC: Immunohistochemistry
IL: Interleukin
LPS: Lipopolysaccharide
M1-CM: M1 macrophage conditioned medium
MBP: Myelin basic protein
NFκB: Nuclear factor-κB
PNS: Peripheral nervous system
PGP9.5: Protein gene product 9.5
p-AKT: Phosphorylated protein kinase B
p-PI3K: Phosphorylated phosphoinositide 3-kinase
p-ERK: Phosphorylated extracellular signal-regulated kinase
PBA: Sodium phenylbutyrate
PBS: Phosphate buffer saline
PARP: Poly-ADP ribose polymerase
PMA: Phorbol 12-myristate 13-acetate
PAGE: Polyacrylamide gel electrophoresis
RGMW: Relative gastrocnemius muscle weight
RIPA: Radioimmunoprecipitation assay
RPMI: Roswell Park Memorial Institute
SCs: Schwann cells
SD rats: Sprague–Dawley rats
SDS: Sodium dodecyl sulfate
TLR: Toll-like receptors
THP-2: Human monocytic cells
TNFα: Tumor necrosis factor
WD: Wallerian degeneration
