# Supplementary figures and images for: Sodium phenylbutyrate inhibits Schwann cell inflammation via HDAC and NFκB to promote axonal regeneration and remyelination
Source: J Neuroinflammation. 2021 Oct 16;18:238. doi: 10.1186/s12974-021-02273-1 (PMC8520633; doi:10.1186/s12974-021-02273-1)

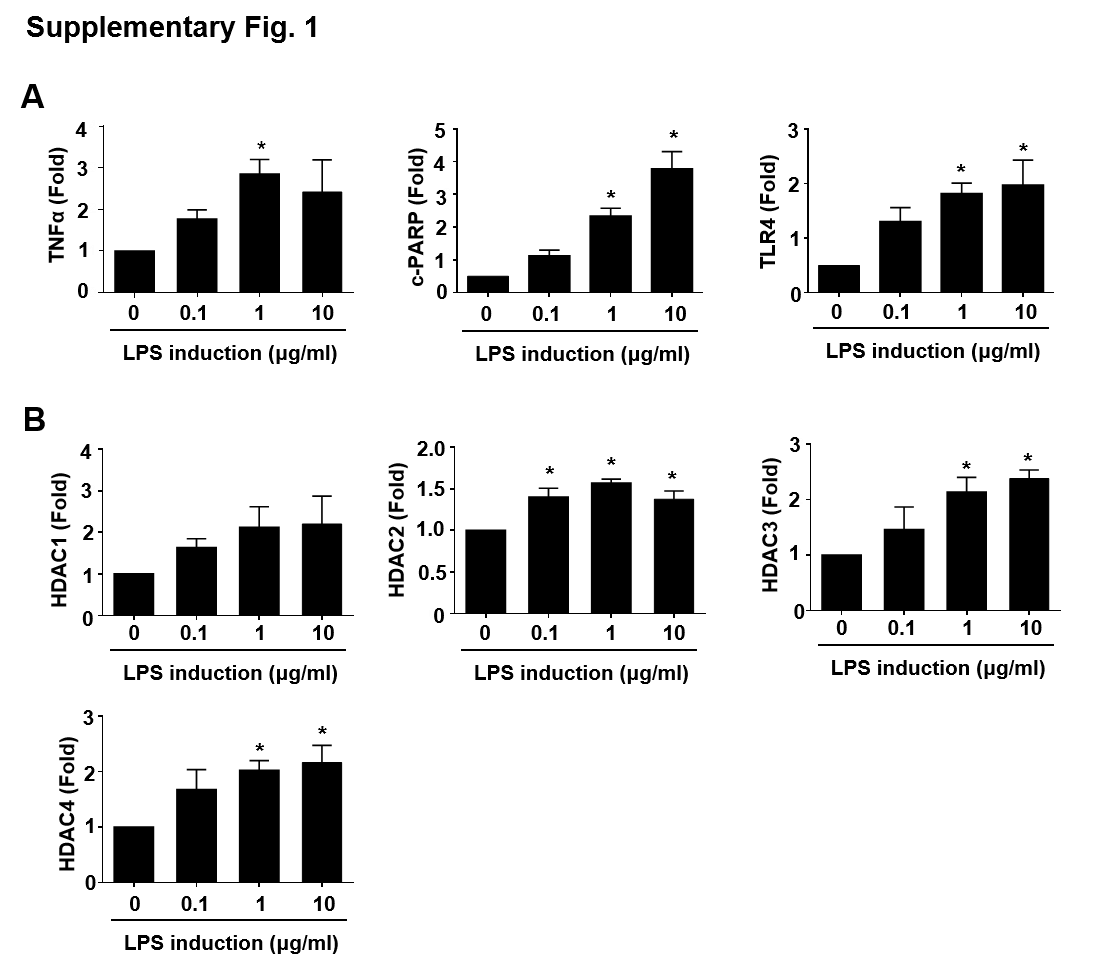

Supplement: Supplementary file 1 — Additional file 1: Figure S1. LPS-induced inflammation and the activation of TLR4 affect the Class I and Class IIa HDAC expression profile. (A) Quantification of the western blot data from Fig. 1. (B) for the expression levels of TNFα, c-PARP, and TLR4 showed an increase in dose-dependent manner after 24 h treatment with LPS. (B) Quantification of western bot data from Fig. 1 (C) shows a change in the expression pattern of HDACs. n = 4. Significance was assessed by one-way ANOVA. The data are presented as the mean ± SEM. *p < 0.05 versus 0 μg/ml LPS induction. LPS: lipopolysaccharide; TNFα: tumor necrosis factor α; c-PARP: cleaved poly (ADP) ribose polymerase; TLR4: Toll-like receptor 4; HDAC: histone deacetylase; ANOVA: analysis of variance; SEM: standard error of the mean. [file 12974_2021_2273_MOESM1_ESM.tif]

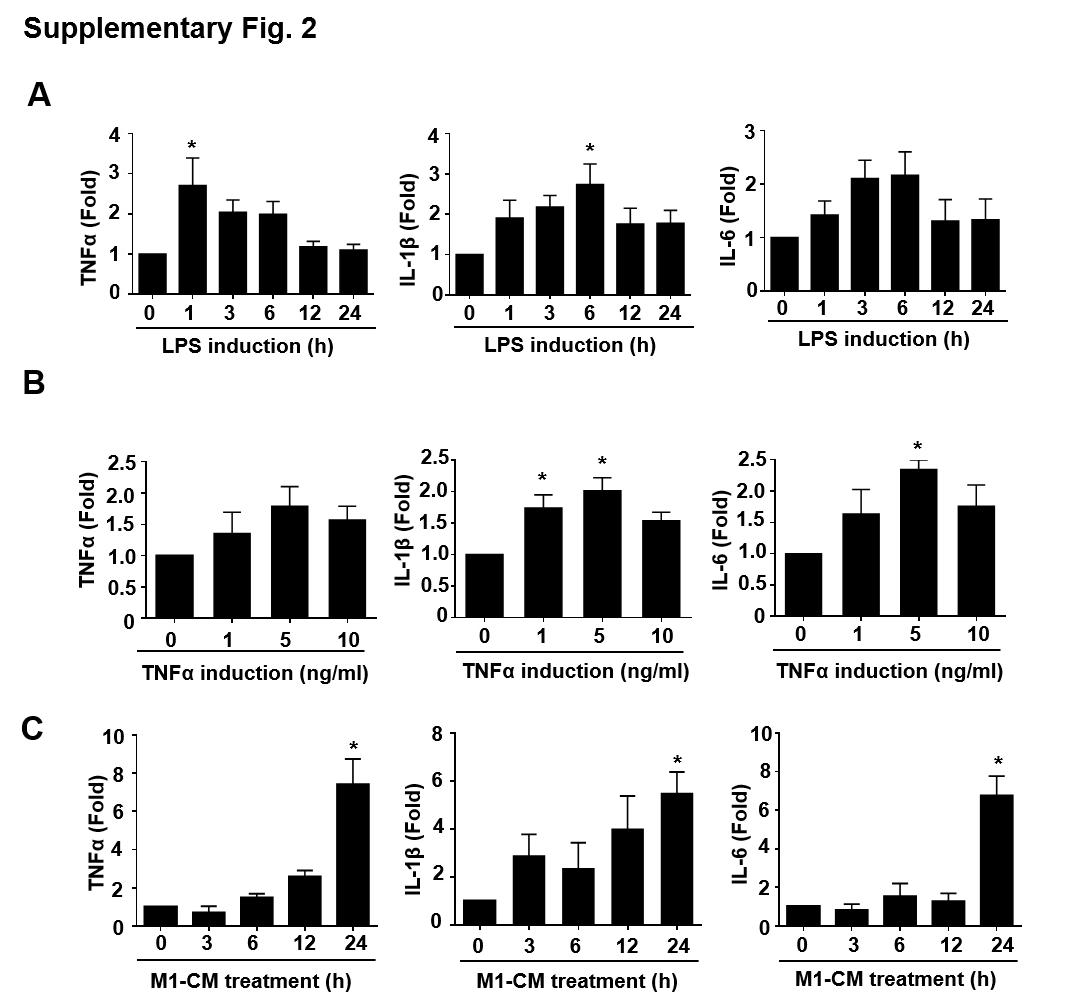

Supplement: Supplementary file 2 — Additional file 2: Figure S2. The mRNA expression of inflammatory cytokines in RT4 SCs in response to various inflammation inducers. (A) qRT-PCR results show changes in the mRNA expression profile of TNFα, IL-1β, and IL-6, in a time-course manner after induction with 1 μg/ml LPS. (B) Increase in levels of inflammatory cytokines in a dose-dependent manner after induction of cells with TNFα for 24 h. (C) Treatment with M1-CM for 24 h significantly increased the mRNA expression levels of TNFα, IL-1β, and IL-6. n = 4. Significance was assessed by one-way ANOVA. The data are presented as the mean ± SEM. *p < 0.05 versus 0 h of LPS treatment in (A). *p < 0.05 versus 0 μg/ml TNFα in (B). *p < 0.05 versus 0 h M1-CM treatment in (C). SCs: Schwann cells; LPS: lipopolysaccharide; TNFα: tumor necrosis factor α; qRT-PCR: quantitative real-time polymerase chain reaction; IL: interleukin; M1-CM, macrophage M1 conditioned media; ANOVA: analysis of variance: SEM: standard error of the mean. [file 12974_2021_2273_MOESM2_ESM.tif]

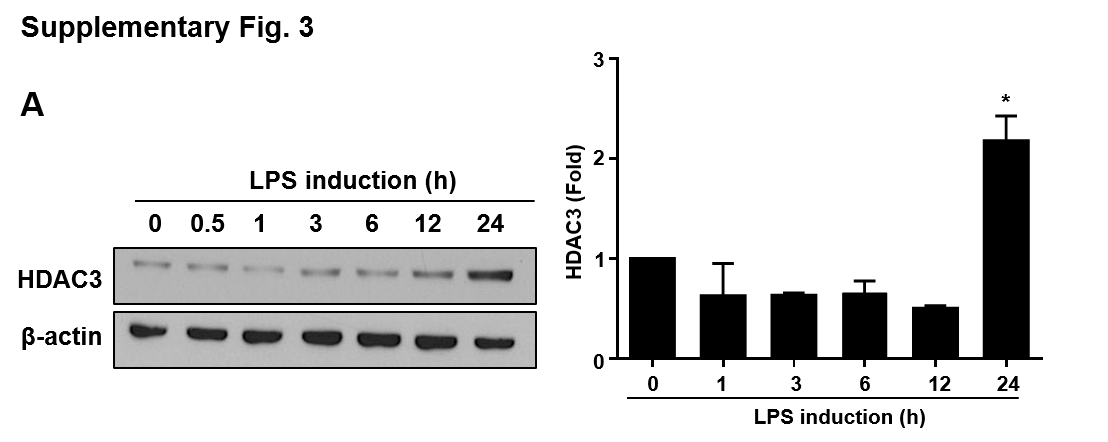

Supplement: Supplementary file 3 — Additional file 3: Figure S3. LPS induces HDAC3 activation after 24 h. (A) Western blot analysis and quantification showing the expression time for HDAC3 after LPS induction in RT4 SCs. n = 4. Significance was assessed by one-way ANOVA. The data are presented as the mean ± SEM. *p < 0.05 versus 0 h LPS induction. #p < 0.05 versus 1 h of LPS treatment. LPS: lipopolysaccharide; HDAC: histone deacetylase; SC: Schwann cell; ANOVA: analysis of variance; SEM: standard error of the mean. [file 12974_2021_2273_MOESM3_ESM.tif]

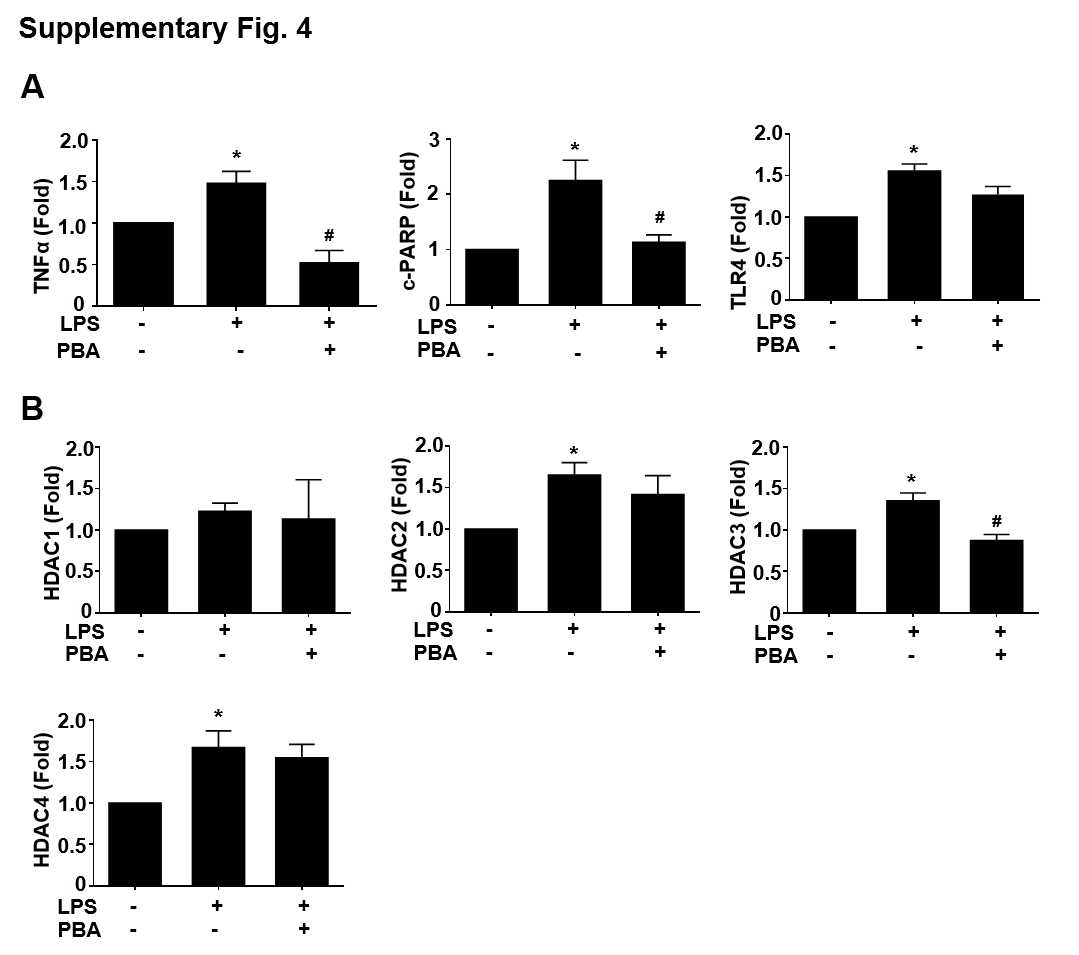

Supplement: Supplementary file 4 — Additional file 4: Figure S4. LPS-induced inflammation, the activation of TLR4, and HDAC modulation are altered by PBA treatment in RT4 SCs. (A) Quantification of western blot data from Fig. 4 (A) for the expression levels of TNFα, c-PARP, and TLR4 revealed a decrease in their expression following LPS and PBA co-treatment for 24 h. (B) Quantification of western blot data from Fig. 4 (C) shows the reduced expression of HDAC3 with LPS and PBA co-treatment. n = 4. Significance was assessed by one-way ANOVA. The data are presented as the mean ± SEM. *p < 0.05 versus no LPS and PBA. #p < 0.05 versus only LPS. PBA: sodium phenylbutyrate; LPS: lipopolysaccharide; SCs: Schwann cells; TNFα: tumor necrosis factor α; c-PARP: cleaved poly (ADP) ribose polymerase; TLR4: Toll-like receptor 4; HDAC: histone deacetylase; ANOVA: analysis of variance: SEM: standard error of the mean. [file 12974_2021_2273_MOESM4_ESM.tif]
